# The C-terminal domain of connexin43 modulates cartilage structure via chondrocyte phenotypic changes

**DOI:** 10.18632/oncotarget.12197

**Published:** 2016-09-22

**Authors:** Raquel Gago-Fuentes, John F. Bechberger, Marta Varela-Eirin, Adrian Varela-Vazquez, Benigno Acea, Eduardo Fonseca, Christian C. Naus, Maria D. Mayan

**Affiliations:** ^1^ CellCOM-SB Research Group, Instituto de Investigación Biomédica de A Coruña (INIBIC), CH-Universitario A Coruña (XXIAC), University of A Coruña, Servizo Galego de Saúde (SERGAS), 15006 A Coruña, Spain; ^2^ Department of Cellular and Physiological Sciences, The Life Sciences Institute, University of British Columbia, V6T 1Z3 Vancouver, British Columbia, Canada

**Keywords:** articular cartilage, connexin43, chondrocyte, osteoarthritis, C-terminal domain of Cx43

## Abstract

Chondrocytes in cartilage and bone cells population express connexin43 (Cx43) and gap junction intercellular communication (GJIC) is essential to synchronize cells for coordinated electrical, mechanical, metabolic and chemical communication in both tissues. Reduced Cx43 connectivity decreases chondrocyte differentiation and defective Cx43 causes skeletal defects. The carboxy terminal domain (CTD) of Cx43 is located in the cytoplasmic side and is key for protein functions. Here we demonstrated that chondrocytes from the CTD-deficient mice, K258stop/Cx43KO and K258stop/K258stop, have reduced GJIC, increased rates of proliferation and reduced expression of collagen type II and proteoglycans. We observed that CTD-truncated mice were significantly smaller in size. Together these results demonstrated that the deletion of the CTD negatively impacts cartilage structure and normal chondrocyte phenotype. These findings suggest that the proteolytic cleavage of the CTD under pathological conditions, such as under the activation of metalloproteinases during tissue injury or inflammation, may account for the deleterious effects of Cx43 in cartilage and bone disorders such as osteoarthritis.

## INTRODUCTION

Chondrocytes and bone cells express different types of connexins (Cxs) and communicate and respond to different stimuli through gap junction (GJ) channels and hemichannels (HCs) [1–3]. A HC is formed by six Cxs and allows the direct communication between the cell and the extracellular environment, including the extracellular matrix (ECM). On the other hand, direct intercellular communication is mediated by GJ channels, which are composed of two HCs, one provided by each adjacent cell [4]. Connexin43 (Cx43) is the most widely expressed GJ protein and is associated with a number of pathological conditions, including skin, bone, cartilage, cardiac and neurological disorders [5, 6]. Several studies have demonstrated that Cx43 has junction-independent functions, many of which can be attributed to the carboxy terminal domain (CTD) [7–10]. A fragment of 20 KDa in size, corresponding to part of the CTD has been described and characterized in different human cell lines and primary cells. In fact the CTD fragments have been associated with different functions and disorders [11–15].

Cx43 is the most abundant GJ protein expressed in chondrocytes and bone cells [1, 2, 16]. Mutations in the Cx43 gene sequence lead to the development of several diseases and syndromes, which include bone and cartilage diseases [17, 18]. Mice lacking Cx43 die soon after birth due to cardiac malformations, nevertheless, neonatal bones exhibit delayed ossification and deficient mineralization [17, 19]. In addition, recent reports have convincingly demonstrated the involvement of Cx43 in the pathogenesis of degenerative joint disorders including osteoarthritis (OA) [1, 20–23]. Cx43 plays key roles in bone and cartilage development, structure and function [16]. However, several aspects of Cx43 functions in cartilage and skeletal tissue are still unclear.

The CTD of Cx43 is located on the cytoplasmic side of the plasma membrane and has been implicated in the regulation of GJ life cycle and in the gating of the channels (HCs and GJs) [24–26]. The CTD interacts with several proteins, undergoes different posttranscriptional modifications and contains several potential signalling motifs that regulate channel gating and channel-independent functions of Cx43 [24, 27]. It has been reported that the CTD is as effective as the full-length Cx43 in inhibiting cell proliferation in cancer cell lines [28] and can be translocated to the nucleus and regulate gene expression, differentiation and cell growth [29].

This study reveals that CTD deficiency in mice alters the structure of cartilage and the normal phenotype of the chondrocyte. We found that CTD-deficient chondrocytes have decreased ability to communicate through GJ channels, defective cellular proliferation and decreased levels in the synthesis of the components of ECM, suggesting that the CTD is essential to ensure coordination of cellular activities and normal properties of cartilage. Together with other reports [30, 31], our study in mice suggests that previous findings described for Cx43 in joint tissues may be due to alterations in the CTD more than full-length Cx43, thereby opening new avenues to understanding the pathophysiological mechanisms and providing new insights in order to treat bone and cartilage diseases.

## RESULTS

**Figure 1:**
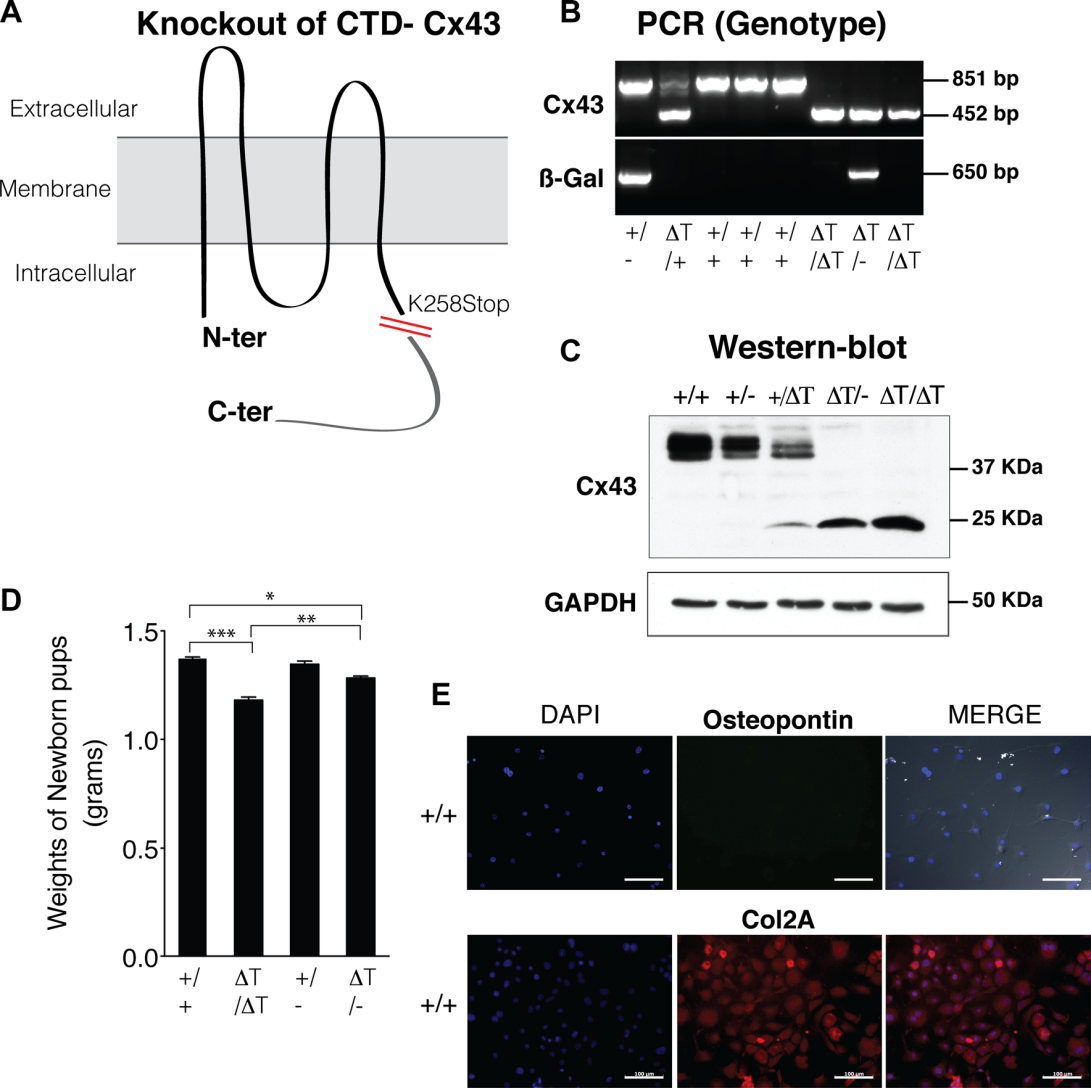
CTD-deficient cells display altered properties in their cell behaviour and phenotype (**A**) CTD-deficient mice (ΔT) express a carboxyl-terminal truncated Cx43 (K258Stop) instead of the WT Cx43 isoform [32]. (**B** and **C**) Mouse genotyping was performed by PCR and confirmed by western blot using an antibody raised against the N-terminal region of Cx43, in order to detect the full length (+/+ and +/−) and the truncated CTD mutants (2–4 day old mice). Cx43K258stop protein has a prolonged half-life time [32]. ß-Gal image represents the PCR amplicons for the ß-galactosidase that was inserted into the Cx43 allele to replace it. (**D**) CTD-mutant pups showing smaller body size than age and sex-matched wild type animals, were weighed as soon as they were born to avoid loss of water due to dehydration. Data is shown as mean ± S.E.M. (*n* = 9). (**E**) Osteopontin (marker of bone cells) and collagen type II (Col2A, chondrocyte specific marker) were determined by immunofluorescence on mouse primary knee articular chondrocytes isolated from wild-type mice. Scale bar represents 100 *μ*m.

In the CTD-deficient mice, the coding region of Cx43 gene was replaced with a Cx43 gene sequence lacking the last 125 amino acids residues of the CTD (K258 stop mice)[32] (Figure 1A). Homozygous K258 stop mice (genotype referred to as ΔT/ΔT) die shortly after birth owing to a disruption in epidermal differentiation [32]. On the other hand Cx43 knockout mice (referred to as −/−) die in the early postnatal period with cardiac defects and from neonatal pulmonary outflow obstruction [33]. To circumvent these difficulties we used newborn and 2–4 day old mice. Cartilage and primary chondrocytes of wild type (+/+), Cx43/KO (+/−), K258stop/KO (ΔT/−) and K258stop/K258stop (ΔT/ΔT) mice were subjected to the study. Mouse genotyping was achieved by PCR using DNA extracted from ear tissue and further tested by western blot using whole cell lysate from primary chondrocytes (Figure 1B, 1C). Under macroscopic observation, the gross morphology of CTD-deficient mice (ΔT/ΔT and ΔT/−) showed shorter body as compared with the control mice (+/+ and +/−) and in concordance with observations reported by other authors [32] (Figure 1D and Supplementary Figure S1).

The chondrocytes were isolated under a light microscope in order to separate the cartilage from the rest of the tissues in the joint. Immunofluorescence experiments demonstrated that primary chondrocytes maintained their phenotype showing high expression of collagen type II (Col2A) and lack of the bone marker osteopontin (Figure 1E).

**Figure 2:**
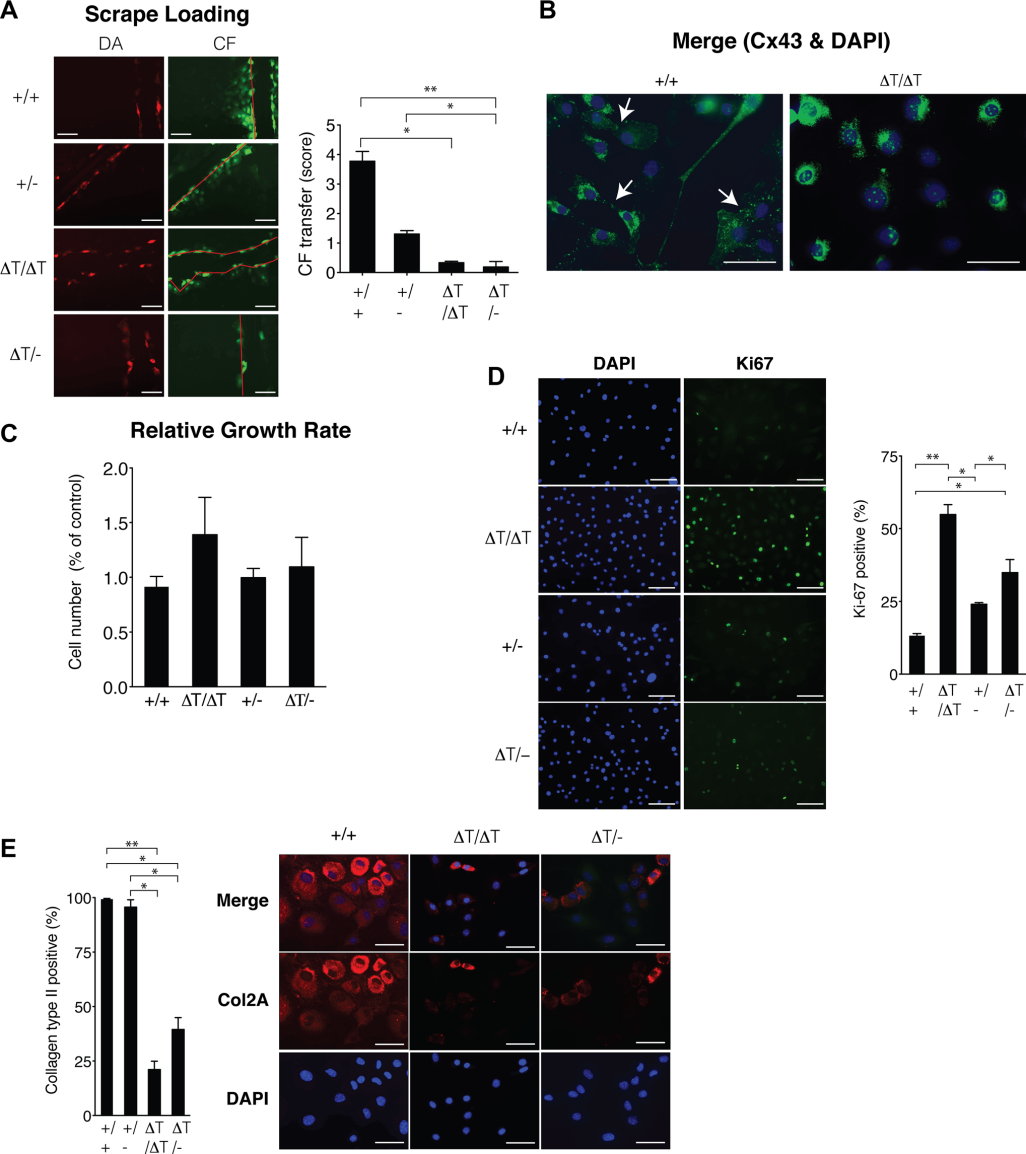
CTD-deficient cells displayed altered cell behaviour (**A**) Primary chondrocytes from 2–4 day old mice were subjected to scrape load assays in the presence of carboxyfluorescein (green) to study the GJIC. Extent of dye transfer was quantified by counting the cells from the scrape line (Dextran Alexafluor 568 positive cells, red) to the dye front for carboxyfluorescein. The score represents the number of contacted cells into which the carboxyfluorescein was transferred per scrape line (number of red cells). Mean ± S.E.M. (*n* = 4 for +/+ and ΔT/−, *n* = 5 for +/− and *n* = 9 for ΔT/ΔT). Scale bars = 100 μm. (**B**) Cellular localization of Cx43 was studied by immunofluorescence. Scale bar represents 50 *μ*m. (**C** and **D**) Cell proliferation was evaluated by monitoring changes in the total count with time (24, 48, 72 and 144 h) using an automated cell counter as explained in methods (C) and by counting the number of positive cells for Ki67 (D). Mean ± S.E.M. (*n* = 5). Scale bar represents 100 *μ*m (**E**) Collagen type II (COL2A) protein levels in primary chondrocytes were determined by immunofluorescence. Scale bar represents 50 *μ*m. CTD-deficient chondrocytes showed significantly less immunofluorescent staining and positive cells. *p* values shown were determined by Mann Whitney test or the Kruskal-Wallis test with Dunn's Multiple Comparison test. *p < 0.05; **p < 0.01; ***p < 0.001.

**Figure 3:**
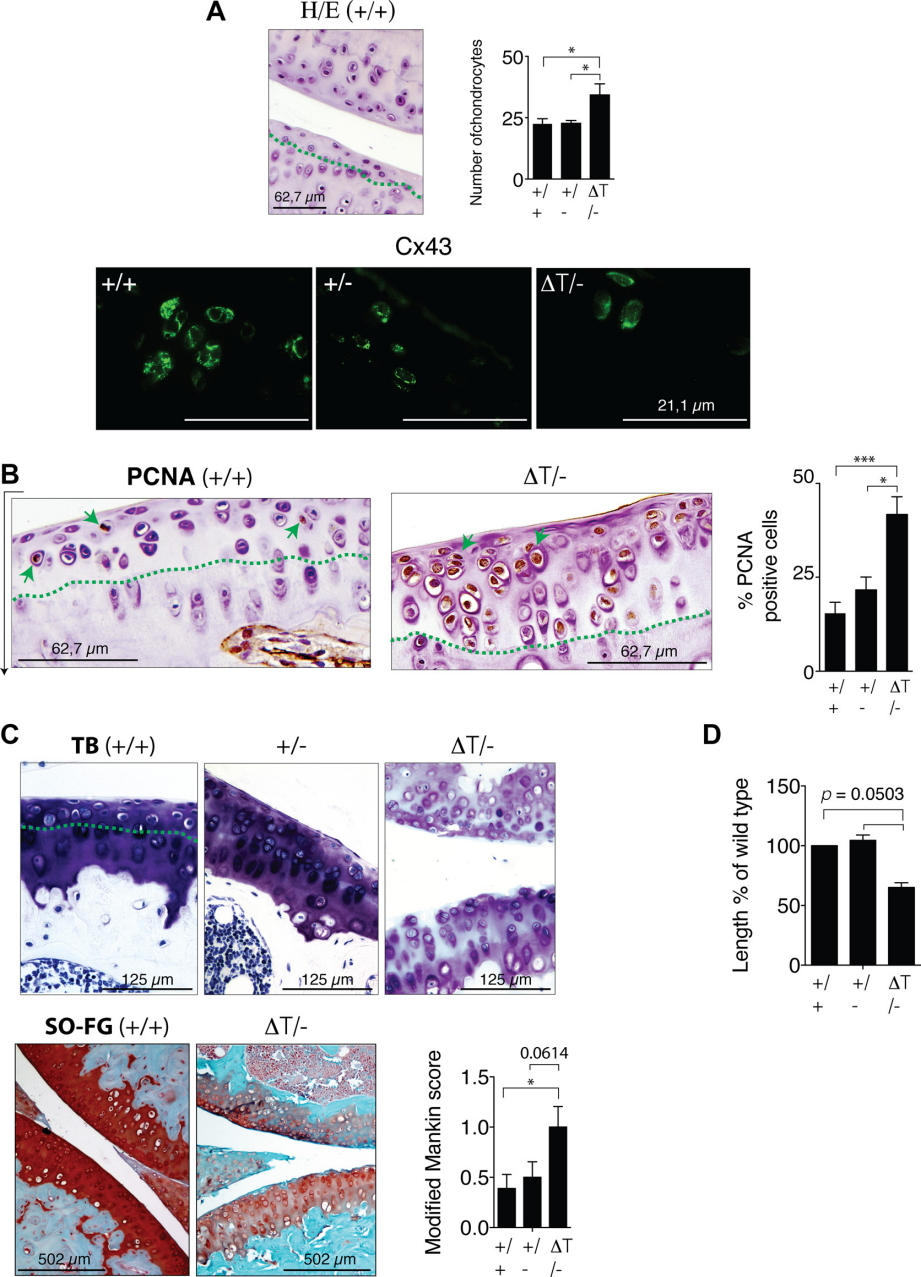
CTD-deficient mice have changes in the ECM composition of cartilage (**A**) Cartilage staining with HE of wild-type (+/+), (+/−) and CTD-deficient mice (ΔT/−) showed a significant increase in the number of chondrocytes in the cartilage of CTD-mutant mice (eight month old mice). The increase in cellularity was studied in the noncalcified cartilage interface above the tidemark (green dashed line). A representative image is shown. Mean ± S.E.M. (*n* = 21 for +/+, *n* = 10 for +/− and for ΔT/−). Below, cellular localization of Cx43 and CTD-truncated Cx43 in cartilage was studied by immunofluorescence. (**B**) IHC analysis using an anti-PCNA antibody. Mean ± S.E.M. (*n* = 24 for +/+, *n* = 10 for +/− and *n* = 17 for ΔT/−). (**C**) Safranin-O fast green, toluidine blue staining of knee joints and modified Mankin score [77, 78] (below *n* = 9), eight month old mice. CTD-deficient mice showed reduced staining in the femoral condyles and tibial plateaus. The increase in cellularity was also observed in the cartilage area above the tidemark. (**D**) Diagram showing the relative length of long bones (tibia and femur) of newborn CTD-deficient mice compared to the wild type. Mice pups on day of birth were used to perform these studies. Mean ± S.E.M. (*n* = 5) *p < 0.05; **p < 0.01; ***p < 0.001, Mann-Whitney test.

Although the CTD-truncated Cx43 can form open gap junctional channels [32, 34–36], scrape loading assays using primary chondrocytes and carboxyfluorescein indicated a significant decrease in the transfer of carboxyfluorescein between CTD-deficient chondrocytes compared with chondrocytes isolated from WT animals (Figure 2A). We have also observed a loss of the localization of the Cx43 in the plasma membrane of chondrocytes that expressed the truncated-Cx43 (Figure 2B and Supplementary Figure S2). In the CTD-deficient chondrocytes (ΔT/ΔT), Cx43 was mainly localized in the cytoplasmic compartments surrounding the nucleus, with a few positive signals for truncated Cx43 on the margin of the cells (Figure 2B and Supplementary Figure S2). A number of studies have reported both pro- and anti-proliferative effects of Cx43 in the regulation of cellular proliferation that depend on the cell, tissue type or microenvironment [37]. Proliferation assays revealed an increase in cellular proliferation in the CTD-deficient chondrocytes (Figure 2C, 2D). Immunohistochemistry (IHC) experiments using primary chondrocytes showed decreased levels of the main chondrocyte marker Col2A in the CTD-deficient chondrocytes (Figure 2E). Consistently, the analysis by staining techniques and IHC assays using sections obtained from *in vivo* cartilage confirmed a significantly increased number (higher than 30%) of chondrocytes in the cartilage of CTD-deficient animals compared with WT, accompanied with changes in the cellular localization of the CTD-truncated Cx43 (Figure 3A). Immunofluorescence experiments using cartilage sections showed altered localization of the truncated-Cx43 in the margin of the lacunae (plasma membrane) of chondrocytes. Cx43 was predominantly located in the margin of the cells (Figure 3A). However CTD-truncated protein was found mainly diffuse in the cytoplasm and around the nucleus with a few positive spots in the margin of the cell, showing the same altered location that was previously observed in CTD-deficient primary chondrocytes in culture (Figures 3A, 2B and Supplementary Figure S2). The changes observed in the number of cells in the cartilage of CTD-deficient animals were confirmed by the observation of a significant increase in the positive staining for the proliferation antigen PCNA by immunohistochemistry assays (Figure 3B).

Col2A together with proteoglycans are the most characteristic components of the cartilage ECM. CTD-deficient chondrocytes showed decreased levels of Col2A (Figure 3C). The staining techniques using the cationic stains Safranin-O fast green (SO-FG) and toluidine blue [38] demonstrated that the CTD-deficient cartilage contain lower levels of proteoglycans detected by loss of strong orange-red staining (SO-FG) and loss of blue staining in the case of TB (Figure 3C). The cartilage structure was studied using a semi-quantitative method. Each section was analysed for abnormalities in cellularity, haematoxylin and eosin (HE) staining, TB staining and SO-FG distribution. The data represent the Mankin score for 9 animals (Figure 3C). Decreasing the K258stop gene dosage (ΔT/−) rescues the lethal epidermal phenotype of double mutant ΔT/ΔT [32]. The very rare mutant with reduced survival [32] allowed us to measure and weigh some newborn mice. CTD-truncated animals weighed significantly less than other genotypes (Figure 1D). The length of the long bones of the mice pups on the day of birth was reduced in mutants compared to control animals (Figure 3D). Female and male ΔT/− mice confirmed these results and were 16.8% and 13.5% smaller, respectively. Furthermore CTD-truncated four months old mice weighed significantly less than other genotypes that contain at least one copy of full-length Cx43 (Supplementary Figure S1). The length of femur and tibia from both legs from female (Supplementary Figure S1A) and male (Supplementary Figure S1B) animals were significantly shorter in the ΔT/− mice.

## DISCUSSION

In the last decades, some of the Cx43 channel-dependent and channel-independent functions have been associated with the CTD more than with the full-length Cx43 protein. The CTD regulates intracellular signalling acting as a scaffold for structural and signalling proteins [20]. As such, CTD-deficient mice offer an experimental system to analyse the role of Cx43 in chondrocytes and cartilage. Our results in mouse models of normal (full length Cx43), heterozygous for the deletion of Cx43, heterozygous for the CTD-truncated Cx43 and CTD-null mice indicate that the CTD plays a significant role in cartilage by controlling the phenotype and normal behaviour of chondrocytes. Chondrocytes have very low rates of cellular proliferation and high metabolic activity in order to synthesise the matrix components and maintain normal cartilage structure and function. The overall reduction in Cx43 (+/−) slightly affects the phenotype, probably due to the reduction in protein levels and GJIC. However mutant chondrocytes (CTD-truncated Cx43) showed a significantly higher rate of cellular proliferation and reduced ability to synthesise the cartilage markers Col2A and proteoglycans together with decreased GJIC. The increase in cellular proliferation changes the number of chondrocytes in the cartilage of mutant mice, reducing the area corresponding to the ECM, thereby potentially altering the mechanical properties of the tissue.

Cx43-mediated cellular communication is highly susceptible to changes in the intracellular and extracellular environment and the CTD is a pivotal player in regulating the chemical gating of Cx43 channels that affect for example to the release of ATP. HCs and GJs are regulated by protein-protein interactions and posttranslational modifications of the CTD sequence. Scrape loading assays indicate that, in chondrocytes, Cx43 requires the integrity of the CTD to properly communicate through GJ channels. Changes in the Cx43 protein localization are consistent with the reduced levels of GJIC, suggesting that in primary chondrocytes, the CTD affects the efficiency of assembly of channels by participating in GJ formation at the membrane and/or in the correct trafficking of Cx43 to the membrane to couple cells through functional GJ channels [39–41]. Changes in GJIC may be responsible for the alterations in cellular proliferation and protein synthesis observed in CTD-deficient chondrocytes. Actually, it has been reported that gradual shortening of the CTD-Cx43 produced a gradual reduction of functional GJs in a paired *Xenopus laevis* oocyte expression system [42]. On the other hand a minimum length of the CTD of Cx32 is required for the formation of functional channels [43]. Efficiency of incorporating channels in the membrane decreases gradually with the progressive shortening of the CTD [43]. Although it has been reported that the CTD-Cx43 is involved in regulating the localization, number and size of the Cx43 plaques [44]. The ΔT/− mice show an increase in the size of the GJ plaques and a reduction in their number in the intercalated disc (myocytes) of ventricular sections [44]. Our results agree with those reports in the sense that truncation of the CTD in mice leads to a decrease in the membrane-positive signals for truncated Cx43 and a significantly reduction in GJIC activity. Yet, our results also show that the lack of part of the CTD affects cellular proliferation and the behaviour of the chondrocytes. Thus, it is possible that other mechanisms are also involved in the phenotypic changes observed in CTD-deficient chondrocytes. Cx43, GJs and the CTD itself are known to participate in the control of cell proliferation. However the effects on cell proliferation depend on the cell type and it is not necessarily related with the activity of the channels [29, 45, 46]. The interplay between the GJIC-dependent and GJIC-independent mechanisms may be involved in the increased levels of cellular proliferation in CTD-deficient chondrocytes with significantly reduced GJIC. The exact mechanisms of the GJIC, Cx43 protein or the CTD fragment on the regulation of cellular proliferation and gene expression remains largely unknown. Although CTD-truncated Cx43 no longer interacts with proteins binding to this fragment, which mediate interactions between GJs and actin cytoskeleton and channel-dependent and independent functions.

Cx43 is a critical protein for bone and cartilage response to loading and unloading as the transmission of mechanical signals also occurs through Cx channels (GJs and HCs) [47–49]. Besides, alterations in chondrocyte proliferation in growth plates contributes to abnormal cartilage growth and bone formation [50]. It is well known that bisphosphonates and other bone/cartilage protecting drugs such as the parathyroid hormone (PTH) require the expression of Cx43 to be effective [51, 52]. Interestingly, several recent reports suggested that signalling mechanisms that altered subchondral bone homeostasis and reciprocal communication (bone-cartilage) play a pivotal role in triggering the degeneration that occurs in cartilage and synovial joints of OA patients [53–55]. Undeniably, the advance in the knowledge of the role of Cx43 and the communication between bone and cartilage will increase our understanding of the pathophysiological mechanisms and will provide new insights in order to treat bone and cartilage related-diseases.

Cx43 deficiency impairs bone formation and healing [56]. However alterations in healing are more pronounced during the mineralization phase of healing where the cartilaginous callus needs to be replaced with areas of new mineralized tissue [57]. The skeleton of the newborn Cx43 null mice (−/−) shows defects in endochondral and intramembranous ossification, resulting in severe dwarfism and reduced bone density [17, 58, 59]. Increased chondrocyte and osteoblast proliferation but alteration in cellular differentiation lead to skeletal abnormalities and less mineralization in pannexin 3 (Panx3) null mice [60–62]. Surprisingly, Panx3 regulates skeletal formation through mechanisms that are distinct from Cx43, promoting the expression of osteogenic genes and Cx43 (GJA1). These authors suggest that Cx43 plays an important role in the maturation stage of osteogenesis while Panx3 regulates bone development in earlier stages. Overexpression of Cx43 since the early stages of OA are accompanied by phenotypic changes and higher rates of cell proliferation [1, 63]. On the other hand, it has been reported that Panx3-deficient mice have decreased risk to suffer degeneration of damaged cartilage [64]. Yet the activity of Panx3 includes the regulation of Cx43 gene expression [60]. Understanding how chondrocytes undergo phenotypic modulation to respond to stimuli and normal homeostasis in articular cartilage matrix turnover will lead to the identification of targets to promote cartilage repair.

The CTD interacts with different proteins that modulate cellular functions such as, for example, the sequestration of β-catenin in the membrane [65]. Our results reveal that a complete absence of the CTD has a negative impact on cartilage structure leading to a significant reduced body size. The abnormal behaviour of the chondrocyte may involve (i) the lack of interaction with signalling molecules such as β-catenin, (ii) alterations of mechanical properties or [38] modifications in the capacity of chondrocytes to respond to different stimuli thought Cx channels. Cx43 connectivity is required for the response to mechanical stimulation [66] and essential to maintain normal bone formation and resorption in order to keep the integrity of the skeleton [67]. It has been recently reported that the CTD is involved in bone acquisition and the removal of the CTD results in decreased cancellous bone mass and reduced anabolic effects of modulators such as PTH [30]. During endochondral ossification, growth plate cartilage is replaced with bone. The reduced body of CTD-deficient mice may be related with the turnover of mineralized growth plate cartilage into bone. It is unclear how this process is regulated, but some authors suggested that occurs in response to mechanical loading and would be regulated by the adjacent bone tissue. The study presented here investigated the role of the CTD in chondrocyte function and cartilage structure. OA patients show evidence for limited calcification of cartilage. Hypertrophy is accompanied by calcification of the normally uncalcified ECM of articular cartilage. The results presented here provide a platform to design new studies that will bring further evidences to understand the role of CTD in chondrocyte matrix mineralization.

Interestingly, such truncation of the CTD occurs in humans under pathological conditions. It was recently suggested that the proteolytic cleavage of the CTD by matrix metalloproteases (MMPs) contribute to acute inflammation during tissue injury [68]. Besides, Cx43 is a MMP-7 substrate whose interaction and cleavage is activated in response to ischemic injury [69]. Cleavage of part of the CTD affects Cx43 protein levels and electrical conduction *in vivo* [69]. Some rheumatology diseases such as OA concur with an increase in cellular proliferation, decrease in the expression of ECM proteins such as Col2A and increased levels of different MMPs. Cx43 protein contains several cleavage sites for different MMPs, including MMP-9, MMP-7 and MMP-2, at several sites of the CTD. Likewise, the MMP proteolysis induced during inflammation, tissue injury or disorders such as OA, may contribute to an increase in cellular proliferation and phenotypic changes associated with Cx43 functional alterations that lead to aberrant tissue repair or progression of the disease.

Recently, the CTD of Cx43 has been described to be a key domain to maintain normal structure and mechanical integrity of bone [30, 31]. Our study suggests that some of the findings related to the roles of Cx43 in cartilage may be due to alterations in the CTD more than full-length Cx43, thereby opening new avenues for the study and understanding of the functions of Cx43 in chondrocytes under normal and pathological conditions. Cx43 has been identified as a regeneration-associated gene. The results reported here demonstrate that the CTD of Cx43 protein plays pivotal roles in normal chondrocyte function and cartilage structure. Some compounds such as Cx mimetic peptides with conserved homology to the cytoplasmic domains of Cx43 are being considered as candidates to improve the rate of wound repair in connective tissues [70–74]. The potential therapeutic implications of novel tissue repair compounds that modulates the CTD of Cx43 protein is an exciting and promising field for degenerative diseases such as osteoarthritis and other cartilage and bone disorders such as osteoporosis.

## MATERIALS AND METHODS

### Genotyping

Characterization of the murine models under study has been reported and were obtained as previously described [32, 33]. Mice were maintained in an animal facility for 12hr light /dark cycle and provided food and water *ad libitum*. The mice were kept on a C57Bl/6J background. All breeding and animal procedures were approved by the University of British Columbia Animal Care Committee and performed in accordance with the guidelines established by the Canadian Council on Animal Care. Genotyping of the mice was performed using ear tissue that was collected after the sacrifice of the mice. For DNA extraction, ear tissue was processed with proteinase K (Proteinase K Solution RNA Grade, Ambion, Life Technologies, USA) during 16–18 hours at 58°C, then it was precipitated with isopropanol (A-416, Fisher Scientific, USA) and washed with ethanol 70% (BP2814, Fisher Scientific, USA). The pellet was dried at 37°C before resuspending with MilliQ water (Millipore, USA). The PCR was carried out in a thermocycler 1720 Thermal Cycles (Applied Byosistems, USA) with the following reaction: 2.5 μl PCR buffer 10×, 1 μl 50 mM MgCl2, 0.5 μL 10 mM dNTPs, 0.5 μl forward and reverse primers (Life Technologies, USA) (Cx43 - 5′ GCATCCTCTTCAAGTCTGTCTTCG 3′ and 3′ CAAAACACCCCCCAAGGAACC 5′ - and β-Gal - 5′ GGCATACAGACCCTTGGACTCC 3′ and 3′ TGCGGGCCTCTTCGCTATTACG 5′), 0.25 μl Platinium Taq (Life Technologies, USA) and 2 μl DNA sample up to 25 μl final volume. After the amplification the samples were loaded into an agarose gel and it was displayed in an AlphaImager TM 4300 camera (Alpha Innotech, USA).

The murine models under study have been previously described [32, 33]. The study was carried out with new born and 2–4 day old pups, and 4 and 8 month old mice. Homozygous K258 stop mice (genotype referred to as ΔT/ΔT) die shortly after birth owing to a disruption in epidermal differentiation [75]. On the other hand Cx43 knockout mice (referred to as −/−) die in the early postnatal period with cardiac defects and from neonatal pulmonary outflow obstruction [33]. Decreasing the K258stop gene dosage (ΔT/−) rescues the lethal epidermal phenotype of double mutant ΔT/ΔT [32, 76]. For the preparation of femur and tibia bones for measurement, mice were euthanized with 120 mg/kg of sodium pentobarbital followed by removal of the back legs. The tissue was removed from the bones and the tibia and femur were separated and the length of each bone measured with a digital calliper to one decimal point.

### Histopathological assays

Mice knee joints were taken to analyse the articular cartilage morphology and structure. Three histological stains were performed on articular cartilage sections: haematoxylin-eosin (HE), Safranine O fast green (SF-FG) and toluidine blue [38]. Mice knee joints were placed inside of cassettes (Tissue Teck, Netherlands), fixed with 2% paraformaldehyde in phosphate-buffered saline (PBS) for 16–18 hours and rinsed in 70% ethanol for 10 minutes, followed by embedding in paraffin (Merck, Germany) with the STP 120 Tissue processor (Myr, Spain). To make the paraffin blocks the Leica EG1150 (Leica Microsystems, Germany) was used. The microtome (Leica RM2155, Leica Microsystems, Germany) was used to prepare 4 μm thick paraffin sections which were placed onto cover slips and dewaxed at 60°C for 10 minutes, cleared with xylol (Panreac Química, Spain) for 10 minutes, hydrated in descending ethanol solutions (100°, 96°, 70°) and distilled water. Coverslips were immersed in Harris' haematoxylin for 5 minutes, exchanged with tap water until the water was clear and then immersed with eosin for 5 minutes, dehydrated in ascending ethanol solutions (96°, 100°) and cleared with xylol. To performed the TB stain the sections were immersed in TB 0.2% in acetate buffer (30 ml solution A (2.7 g sodium acetate in 100 ml distilled water) and 90 ml of solution B (1.1 ml acetic acid 0.5 M in 100 ml de distilled water) pH 4.2 for 2–5 minutes, rinsed with the acetate buffer and then with a 4% ammonium molybdate solution. For SF-GO stain Safranine O was diluted in 50% ethanol and the cover slips were immersed for 30 minutes, rinsed with distilled water followed by four quick immersions in fast green. All cover slips were mounted with DePex (SERVA, Germany) and the samples were photographed by using an Olympus BX61 microscope using a DP71 digital camera (Olympus), and the AnalySISD 5.0 software (Olympus Biosystems, Germany). Articular cartilage of mice was scored using the modified Mankin score following the *Glasson S.S. et al.* method with modifications from *Chambers et al*. [77, 78]. Each sample was analysed for abnormalities in cellularity, haematoxylin and eosin staining, toluidine blue staining, Safranin-O fast green distribution, erosion, and fibrillations. The value of the corresponding grade was calculated using the average punctuation obtained after scoring each sample and group.

### Isolation and primary culture

The mice from each litter (2–4 days old) were sacrificed at the same time under general anesthesia. After sacrificing mice all procedures were made in a sterile flow hood. Mice were sprayed with ethanol 70% and placed in face-up position on a paper-covered cork plate and the anterior legs were fixed using needles in the sterile flow hood. Skin and soft tissues from the hind legs were removed using scissors and forceps. The knee joint was cut with a scalpel and placed on petri dishes in order to isolated femoral condyles and tibial plateau using a scalpel. The articular cartilage was placed in a new petri dish with PBS for a quick wash and cut in small pieces which were placed into a 15 ml tube that was put in ice while the following mouse was processed. When all litters was processed, the articular cartilage collected was incubated with trypsin-EGTA (25300-062, Invitrogen, USA) for 10 minutes at 37°C with shaking and then was incubated with collagenase IV (C5318, Sigma-Aldrich, USA), 2 mg/ml, diluted in DMEM supplemented with 5% FBS (SH30396.03, Hyclone, USA) and 1% penicillin-streptomycin (P4333, Invitrogen, USA) for 14 hours at 37°C with shaking. The articular cartilages processed with collagenase IV solution were filtered with 0.45 μm strains and centrifuged at 1500 – 2000 rpm for 5 minutes at room temperature. The cells were counted on a hemocytometer and were seeded on plates with DMEM supplemented with 10% FBS and 1% penicillin-streptomycin.

### Immunofluorescence techniques and IHC analyses

The cells were seeded on sterile round cover slips (12-545-81, Fisher Scientific, USA), which previously were coated with poly-lysine (P4707, Sigma, EEUU), and were cultured to 80% confluence. For immunofluorescence techniques the antibodies against Ki67 (1:400 dilution, rabbit anti-Ki67, 556003, BD Biosciences, USA), Col2A (1:10, goat anti-collagen type IIsc-7764, Santa Cruz Biotechnology, USA), Cx43 (N-terminus) (1:25, rabbit anti-Cx43Nterminus, AP1541b, Abgent, USA), CTD-Cx43 (1:25, mouse anti-Cx43, A6219, Sigma-Aldrich, USA), osteopontin (1:100, goat anti-osteopontin, PA1-25152, ThermoScientific, USA) and fibronectin (1:400, mouse anti-fibronectin, F6140, Sigma-Aldrich, USA) were used. The cells were fixed with cold methanol at 4°C for 10 minutes and rinsed with PBS 4 times for 5 minutes. The cover slips were placed into the wells of a 12 well-plates, treated with blocking solution (2% BSA, 15260-037, Invitrogen, USA), Triton X-100 (13021, Sigma-Aldrich, USA) diluted in PBS) for 30 minutes, rinsed with PBS and incubated with the primary antibody for 1 hour at room temperature with shaking. After rinsing 4 times for 10 minutes at room temperature with shaking, the chondrocytes were incubated with the respective secondary antibody (goat anti-rabbit Alexafluor 488, goat anti-mouse IgM Alexa fluor 488, Donkey anti-goat Alexa 568, respectively, at 1:500 dilution supplied by Invitrogen, USA) for 1 hour at room temperature with shaking, rinsed with PBS 4 times at room temperature with shaking and mounted with Prolong^®^ Gold Antifade Reagent with DAPI (P36931, Life Technologies, USA). Samples were analysed by using AXIOCAM MRm ZEISS - HBO100 (Zeiss, Germany) with the Axioplan 2 (Zeiss, Germany) with Axiovison 4.6 (Zeiss, Germany). Controls in the absence of primary antibodies were routinely performed and yielded no signals.

For IHC assays a microtome (Leica RM2155, Leica Microsystems, Germany) was used to prepare 4 μm thick paraffin sections which were dried at 37°C and dewaxed at 60°C for 10 minutes, immersed in xylol for 10 minutes, hydrated with descending ethanol (100°, 96°) for 5 minutes and distilled water. For the IHC the antibody against PCNA (1:100, NA03, Calbiochem^®^, Germany) was used. To block the endogenous peroxidase, hydrated sections were treated with peroxidase blocking solution (Dako, Denmark) for 10 minutes at room temperature and rinsed with PBS. Primary antibody was incubated for 1 hour at room temperature, rinsed with PBS and incubated 1 hour with Polymered Envision plus polyclonal kit Envision™ Detection Systems Peroxidase/DAB, Rabbit/Mouse (Labelled polymer-HPRT, Dako, Denmark) at room temperate, rinsed three times with PBS and incubated with diaminobenzidine in hydrogen peroxide (1:50 dilution) for 2 minutes to reveal the reaction, rinsed with distilled water, counterstained with Gill's haematoxylin (Merck, Germany), dehydrated with ascending ethanol (70°, 96°, 100°) cleared with xylol and mounted with DePex. Samples were photographed by using an Olympus BX61 microscope using a DP71 digital camera (Olympus), and the AnalySISD 5.0 software (Olympus Biosystems, Germany)

### Immunoblotting

For immunoblotting the chondrocytes in culture were trypsinized and the pellet was lysed with RIPA Buffer (1% NP-40 Igepal (I3021 Sigma Aldrich USA), 0.5% sodium deoxycholate (PI-89905 Fisher Scientific USA), 0.1% SDS (BP166 Fisher Scientific USA), 1/7 dilution of protease inhibitors (04693159001, Roche, USA) and 1/10 dilution of phosphatase inhibitors (P5726, Sigma-Aldrich, USA) in PBS pH 7.4) and kept at −80°C. Equal protein amounts from primary chondrocytes (40 μg) were analysed by SDS-PAGE. The blot (PVDF blot, Millipore, USA) was activated with methanol and distilled water and equilibrated with a cold solution of 20% methanol in TBS 1× buffer (TBS 10X (Tris-Base 2 mM, NaCl 150 mM pH 7.5) and 0.05% Tween-20% to 1X dilution) and the proteins were transferred to the blot with the Trans-Blot SD Semi-Dry Transfer Cell (Bio-Rad, USA). The blot was blocked with a 5% milk solution in TBS 1× buffer for 1 hour with shaking. The primary antibodies against Cx43 N-Terminus (1:500, rabbit anti-Cx43 N terminus, AP1541b, Abgent, USA) and GAPDH (1:5000, mouse anti-GAPDH, 5G4Mab6c5, Hystest Ltd, Finland) were incubated overnight at 4°C, rinsed with TBS 1× buffer 4 times for 15 minutes, incubated with secondary antibody (Goat anti-rabbit HRP 1/5000 (32260, Invitrogen, USA) and Goat anti mouse HRP 1:10000 (32230, Invitrogen, USA), respectively) and rinsed 4 times for 15 minutes. The blot was treated with ECL Western Blotting Detection Reagent (GeHealthcare, United Kingdom) and exposed to an x-ray film (B Plus- Full Blue, (CLM 5810, Mandel Scientific, Canada). The x-ray film was processed in a developer (LSCW#330, CAN-Med Healthcare, Canada) and a picture was obtained by scanning the film (EPSON Perfection V500 photo, Epson, Canada).

### Proliferation assays

Cellular proliferation was determined by measuring the Ki67 positive cells by immunofluorescence techniques and by using an automated cell counter. Immunofluorescence on Ki67 (mouse anti-Ki67, 1:200 dilution, 556003, BD Biosciences, USA) was performed and the cells were counted (total cells and positive cells) from the imagines taken by Fluorescent microscopy (Axioplan2, and Aviovision 4.6 software, Ziess, Germany). The percentage was made between positive cells and total cells and it was represented in a bar plot.

For the automated cell counter experiment a Beckman Coulter Z1 Coulter™ Particle Counter (Beckman, USA) was used. The same number of chondrocytes was seeded on plates (28 cm2 culture plate) with DMEM supplemented with 10% FBS and 1% penicillin-streptomycin, the measurements were taken at day 1, 3, 4, 6, and 7 after seeding. For each measurement day three plates were seeded, and each plate was measured three times. The average of the data was determined and the growth rate or proliferation index (number as a percent) was calculated to normalize the data in order to avoid possible mistakes on the seeded number of cells.

### Scrape loading

The dye-coupling assay to test the functionality of GJs was performed using carboxyfluorescein (2187, Sigma-Aldrich, USA) and Dextran AlexaFluor 568 (10,000 MW, D22912, Invitrogen, USA) was used as a control. Chondrocytes were seeded on plates (28 cm^2^ culture plate) and cultured until the confluence reached 100%. Cells were rinsed two times with PBS and then two scrapes were made using a scalpel in the presence of 0.5% (w/v) carboxyfluorescein and 0.5% (w/v) Dextran Alexafluor 568 (D22912, Invitrogen, USA) in DMEM at room temperature. Cells were incubated for 2 min at room temperature. After washing with a DMEM, dye transfer was captured using AXIOCAM MRm ZEISS - HBO100 (Zeiss, Germany) with Axioplan 2 (Zeiis, Germany) with Axiovision 4.6 (Zeiss, Germany) software. The number of dye-positive cells (carboxyfluorescein transfer) from the cutting site (red/green cells) to the farthest visual uptake of carboxyfluorescein (only green cells) indicates the GJ connectivity between cells. The score was calculated as previously reported [3].

### Statistical analysis

All data are presented as mean ± S.E.M. The significance of difference in the mean values was determined using Mann Whitney test or Kruskal-Wallis test with Dunn's Multiple Comparison test. Significant differences are represented as *p < 0.05; **p < 0.01; ***p < 0.001. Statistical data analysis was performed with Prism (GraphPad software version 5).

## SUPPLEMENTARY MATERIALS FIGURES


