# Supplementary material for: The C-terminal domain of connexin43 modulates cartilage structure via chondrocyte phenotypic changes
Source: Oncotarget. 2016 Sep 22;7(45):73055–67. doi: 10.18632/oncotarget.12197 (PMC5341963; doi:10.18632/oncotarget.12197)
Supplement: Supplementary file 1 [file oncotarget-07-73055-s001.pdf]

The C-terminal domain of connexin43 modulates cartilage structure via chondrocyte phenotypic changes

Supplementary Materials

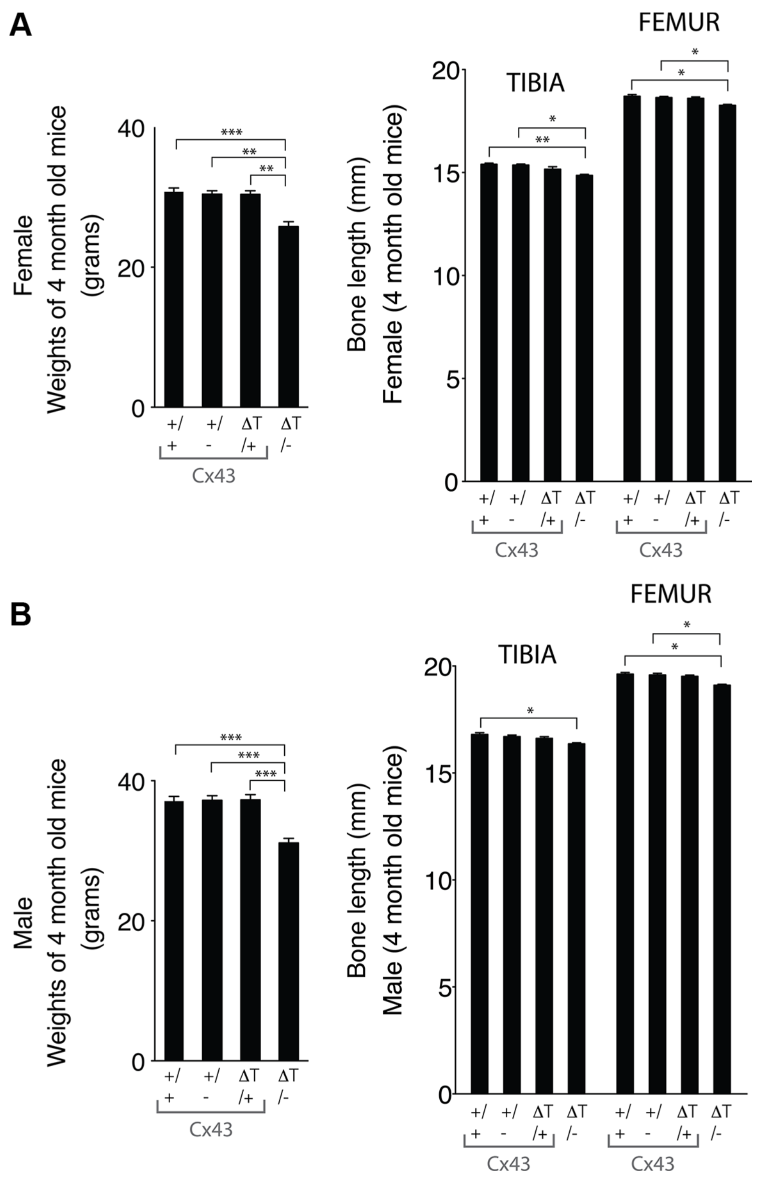

Supplementary Figure S1: CTD-truncated mice (four month old mice) have shortened bone length. (**A** and **B**) Truncated Cx43 genotype mice were smaller than the other genotypes from the same litters. The difference in weight was related with a smaller body without lack of macroscopic fat at visual inspection. The length of each bone was measured using a digital calliper. Mean ± S.E.M. ($n = 5$). $*p < 0.05$; $**p < 0.01$; $***p < 0.001$.

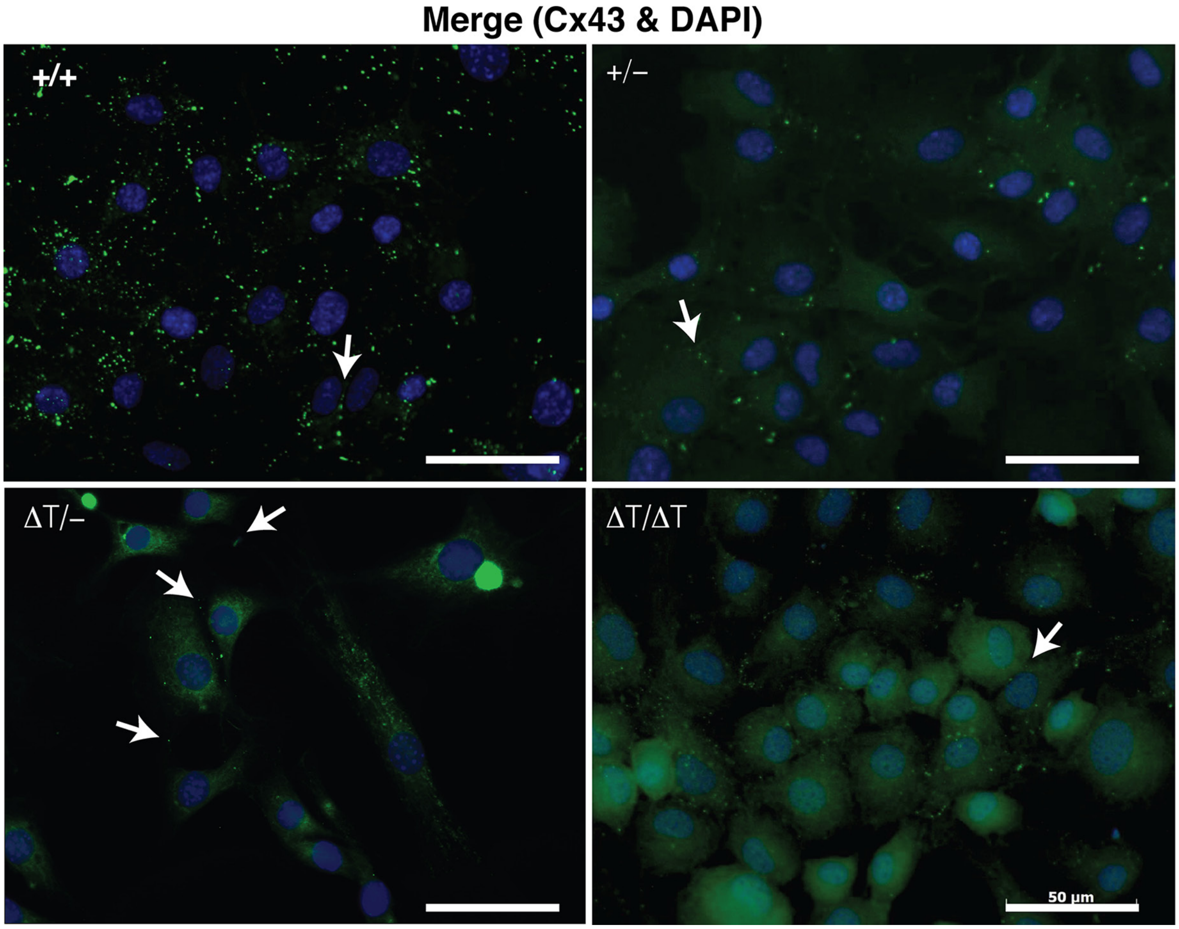

Supplementary Figure S2: Cx43 cellular localization in primary chondrocytes of wild type and truncated Cx43 genotypes (cells isolated from cartilage of 2–4 day old pups) was determined by immunofluorescence. White arrows indicate connexin plaque staining at points of cell-cell contact. Scale bar represents 50 μm.
